# Different response of the oxygen pathway in patients with chronic thromboembolic pulmonary hypertension treated with pulmonary endarterectomy versus balloon pulmonary angioplasty

**DOI:** 10.3389/fcvm.2022.990207

**Published:** 2022-09-27

**Authors:** Zhihui Fu, Xincao Tao, Wanmu Xie, Peiran Yang, Qian Gao, Jinzhi Wang, Zhenguo Zhai

**Affiliations:** ^1^Department of Pulmonary and Critical Care Medicine, Center of Respiratory Medicine, China-Japan Friendship Hospital, Beijing, China; ^2^National Center for Respiratory Medicine, Beijing, China; ^3^Institute of Respiratory Medicine, Chinese Academy of Medical Sciences, Beijing, China; ^4^National Clinical Research Center for Respiratory Diseases, Beijing, China; ^5^Graduate School of Peking Union Medical College, Chinese Academy of Medical Sciences, and Peking Union Medical College, Beijing, China; ^6^Department of Pulmonary and Critical Care Medicine, Quanzhou First Hospital Affiliated to Fujian Medical University, Quanzhou, China; ^7^State Key Laboratory of Medical Molecular Biology, Department of Physiology, Institute of Basic Medical Sciences, Chinese Academy of Medical Sciences, Peking Union Medical College, Beijing, China

**Keywords:** balloon pulmonary angioplasty (BPA), pulmonary endarterectomy (PEA), chronic thromboembolic pulmonary disease, oxygen pathway, effect

## Abstract

**Background:**

Oxygen pathway limitation exists in chronic thromboembolic pulmonary hypertension (CTEPH). Pulmonary endarterectomy (PEA) and balloon pulmonary angioplasty (BPA) are two effective interventions for CTEPH, but their effects and comparison of these two interventions on the oxygen pathway are not well demonstrated.

**Methods:**

CTEPH patients with available pulmonary function test, hemodynamics, and blood gas analysis before and after the interventions were included for comparison of oxygen pathway in terms of lung ventilation, lung gas exchange, oxygen delivery, and oxygen extraction between these two interventions.

**Results:**

The change in the percentage of the predicted forced expiratory volume in the 1 s (−3.4 ± 12.7 vs. 3.8 ± 8.7%, *P* = 0.006) and forced vital capacity (−5.5 ± 13.0 vs. 4.2 ± 9.9%, *P* = 0.001) among the PEA group (*n* = 24) and BPA group (*n* = 46) were significantly different. Patients in the PEA group had a significant increase in their arterial oxygen saturation (from 92.5 ± 3.6 to 94.6 ± 2.4%, *P* = 0.022), while those in the BPA group had no change, which could be explained by a significant improvement in ventilation/perfusion (−0.48 ± 0.53 vs. −0.17 ± 0.41, *P* = 0.016). Compared with patients post-BPA, patients post-PEA were characterized by higher oxygen delivery (756.3 ± 229.1 vs. 628.8 ± 188.5 ml/min, *P* = 0.016) and higher oxygen extraction (203.3 ± 64.8 vs. 151.2 ± 31.9 ml/min, *P* = 0.001).

**Conclusion:**

Partial amelioration of the oxygen pathway limitations could be achieved in CTEPH patients treated with PEA and BPA. CTEPH patients post-PEA had better performance in lung gas exchange, oxygen delivery, and extraction, while those post-BPA had better lung ventilation. Cardiopulmonary rehabilitation may assist in improving the impairment of the oxygen pathway.

## Introduction

Chronic thromboembolic pulmonary hypertension (CTEPH) is considered a severe complication of acute pulmonary embolism with an occurrence of ∼3% (1). Pulmonary endarterectomy (PEA) and balloon pulmonary angioplasty (BPA) are the most effective for CTEPH and have been suggested in the current treatment guidelines (2, 3). Generally, PEA is the recommended treatment for CTEPH patients with operable vascular lesions, while BPA is an alternative option for those not suitable for PEA. The estimated 3-year survival is nearly 90% in CTEPH patients after PEA, compared with 70% of survival in patients without operation (4). Similarly, in a recent meta-analysis study, CTEPH patients were found to have a 97% 3-year survival rate after BPA (5). These encouraging results suggest that PEA and BPA are clearly effective as remedies for the vascular lesions and their fatal consequences in CTEPH.

The oxygen pathway includes multiple complex steps including uptake in the lungs, transportation from alveoli to blood, carrying capacity of the blood, delivery from center to the periphery, extraction of peripheral tissue, and cellular use (6). Impairments in the oxygen pathway can occur in various diseases including heart failure, leukemia, tumors, connective tissue diseases, and respiratory diseases, leading to hypoxia and death (7–12). In patients with CTEPH, occlusion of the pulmonary artery can reduce lung perfusion, causing mismatched ventilation/perfusion and an abnormality in lung function, particularly a decreased lung diffusion capacity (13, 14). Moreover, a recent seminal study found that in addition to the pulmonary gas exchange, multiple steps of the oxygen pathway were defective in patients with CTEPH, including oxygen extraction by peripheral tissues, and pulmonary vascular interventions could partly correct the impaired oxygen pathway (15), in which only 10 patients had available data on the oxygen pathway after intervention. Despite the improved outcomes of CTEPH patients treated with PEA and BPA, the effects of these two interventions on the oxygen pathway are not well demonstrated and have not yet been compared. To better understand the pathophysiological mechanism and to compare the effects of PEA and BPA on the oxygen pathway, we retrospectively collected data, including pulmonary function test, hemodynamics, and blood gas analysis, from CTEPH patients before and after the interventions (PEA or BPA), and different responses of the oxygen pathway in patients treated with PEA versus BPA were revealed.

## Materials and methods

### Patients’ characteristics

**FIGURE 1:**
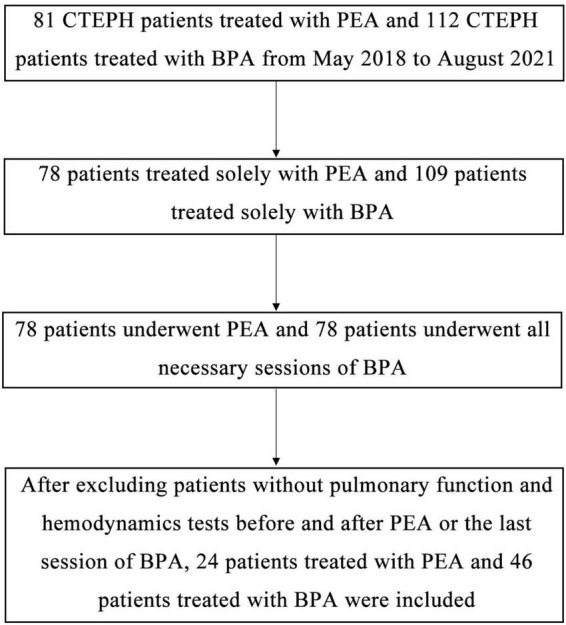
Schematic diagram of the screening process to include or exclude cases. After searching the electronic medical record using ICD-9-CM3, we found 89 patients receiving PEA and 166 patients receiving BPA from May 2018 to August 2021 in our center. After excluding patients without CTEPH, patients receiving both PEA and BPA, patients who have not received all sessions of BPA, and patients without available pulmonary function test before and after intervention, we finally enrolled 24 patients treated with PEA and 46 patients treated with BPA.

Patients with CTEPH who underwent PEA or BPA from May 2018 to August 2021 in our center, were included in this study. Patients in the BPA group should receive all necessary rounds of BPA. Patients who received both PEA and BPA, or those without pulmonary function test and hemodynamics before and after intervention were not included in this study. Data on the pulmonary function test (MasterScreen SES, Vyaire Medical GmbH, China), right heart catheterization (hemodynamics and blood gas analysis), echocardiogram (Vivid E95, GE Healthcare, USA) before and after PEA or the last session of BPA, were retrospectively collected (Figure 1). A multidisciplinary team comprising pulmonary physicians, BPA interventionalists, cardiac surgeons, ICU doctors, and radiologists made the choice of treatments for patients with CTEPH. A refined multi-session BPA was conducted for non-surgical patients according to the previous studies (16). The target of BPA was to achieve an ultimate mean pulmonary artery pressure (mPAP) of less than 25 mmHg and a decrease of 5–10 mmHg in mPAP in each session in our center. Generally, the follow-up pulmonary function test, right heart catheterization, and echocardiogram were carried out simultaneously 6–12 months after the interventions. This study conformed to the principles outlined in the Declaration of Helsinki and was approved by the institutional board and the ethics committee of the China-Japan Friendship Hospital (2021-136-K94).

### Oxygen pathway analysis

In this study, the oxygen pathway was comprised of lung ventilation, lung gas exchange, oxygen delivery, and oxygen extraction. We used the percentage of the predicted forced expiratory volume in the 1 s (FEV1%), forced vital capacity (FVC%), maximum mid-expiratory flow (MMEF%), maximum voluntary ventilation (MVV%), and alveolar ventilation (VA%) to represent lung ventilation. The pulmonary function was tested using MasterScreen (SES, Vyaire Medical GmbH, China) following the American Thoracic Society (ATS) and European Respiratory Society standards (17). Lung gas exchange was assessed as lung diffusion capacity, alveolar partial pressure of oxygen (PalvO_2_), arterial partial pressure of oxygen (PaO_2_), arterial saturation of oxygen (SaO_2_), and alveolar ventilation-to-cardiac output ratio (VA/CO). Lung diffusion capacity was represented by the diffusion capacity of the lungs for carbon monoxide (DLCO) and oxygen (DLO_2_) (≈1.23 × DLCO) (18). PalvO_2_ was calculated using the following equation: PalvO_2_ (mmHg) = 150–1.25 × PaCO_2_ (mmHg), where PaCO_2_ represents arterial partial pressure of carbon dioxide (19). Oxygen delivery (DO_2_) is the total arterial volume of oxygen delivered to peripheral tissues per minute, which was derived from cardiac output (CO, being measured using indirect Fick’s method) and arterial oxygen content (CaO_2_) using the following equation: DO_2_ (ml/min) = CO (L/min) × CaO_2_ (ml/dl) × 10 (19). The oxygen content of blood was calculated using the following equation: O_2_ content (ml/dl) = 1.36 × hemoglobin (g/dl) × SO_2_ + 0.003 × PO_2_ (mmHg), where SO_2_ represents saturation of oxygen and PO_2_ represents partial pressure of oxygen (19). Total oxygen extraction (EO_2_) by the peripheral tissues was defined as the volume gradient of oxygen between arterial blood and mixed venous blood.

### Statistical analysis

Data were analyzed using IBM SPSS STATISTICS 19. Continuous variables were presented as mean ± standard deviation or mean (range), and categorical variables were presented as numbers and percentages. Comparisons of BPA and PEA were using unpaired two-sample *t*-test for continuous variables and χ^2^ or Fisher’s test for categorical variables, where a correction for logic regression analysis was underwent for the analysis of change in the oxygen pathway indices and four parameters [including the interventional method (PEA or BPA), age (0–49, 50–60, and > 60 year), gender (male or female), targeted medications for pulmonary hypertension (yes or no)] were taken into consideration. Comparisons before and after operation were made using paired *t*-test. Pearson correlation coefficient was used to evaluate the correlation between two particular parameters. *P* values < 0.05 were considered statistically significant.

## Results

### Baseline characteristics and oxygen pathway

We enrolled 24 CTEPH patients who received PEA and 46 CTEPH patients who received all sessions of BPA (Figure 1). There were no significant differences between patients who underwent PEA versus BPA in most baseline parameters except in age (Table 1). Patients in the BPA group were significantly older than those in the PEA group (60.3 ± 9.0 vs. 54.0 ± 12.1 year, *P* = 0.024).

**TABLE 1:** Baseline characteristics of CTEPH patients before PEA and BPA.

Characteristics	PEA (*n* = 24)	BPA (*n* = 46)	*P*-value
Age, year	54.0 ± 12.1	60.3 ± 9.0	0.016*
Male	13 (54.2%)	20 (43.4%)	0.455
BMI, kg/m^2^	24.4 ± 3.0	24.6 ± 3.7	0.806
Hemoglobin, g/dl	14.8 ± 1.9	13.9 ± 2.1	0.071
**Functional parameters**			
NT-proBNP, pg/ml	1421 ± 2022	1007 ± 1331	0.307
6MWD, m	367 ± 91	383 ± 90	0.758
WHO-FC			
I-II	9 (37.5%)	26 (56.6%)	0.208
III-IV	15 (62.5%)	20 (43.4%)	
**Echocardiogram**			
RV, mm	46.1 ± 6.5	44.7 ± 7.1	0.428
RV/LV	1.32 ± 0.31	1.34 ± 0.38	0.805
EPASP, mmHg	80.1 ± 18.2	78.5 ± 19.4	0.768
TAPSE, mm	16.3 ± 3.3	17.4 ± 2.8	0.176
S’, cm/s	10.0 ± 2.7	10.6 ± 2.1	0.260
LVEDD, mm	43.9 ± 5.6	42.2 ± 4.3	0.868
PA, mm	32.6 ± 6.8	33.3 ± 6.4	0.697
LVEF, %	69.9 ± 4.9	69.3 ± 5.3	0.641
**Pulmonary function test^#^**			
FEV1, L	2.42 ± 0.81	2.21 ± 0.63	0.244
FVC, L	3.48 ± 0.96	3.09 ± 0.78	0.071
MVV, L/min	73.4 ± 24.7	67.3 ± 19.4	0.270
DLCO, mmol/min/kPa	6.11 ± 1.38	5.83 ± 1.40	0.426
DLCO%, %	71.8 ± 14.0	74.1 ± 14.2	0.533
VA, L	5.20 ± 1.11	4.90 ± 0.93	0.251
MMEF, L/s	1.59 ± 0.83	1.49 ± 0.80	0.639
**Hemodynamics**			
mPAP, mmHg	42.5 ± 11.1	40.4 ± 9.3	0.394
PVR, woods	11.9 ± 5.9	10.2 ± 4.4	0.181
CO, L/min	3.46 ± 1.39	3.34 ± 1.09	0.689
CI, L/min/m^2^	1.86 ± 0.75	1.82 ± 0.53	0.807
PCWP, mmHg	8.8 ± 3.0	9.4 ± 3.2	0.429

Baseline characteristics of CTEPH patients were compared between the PEA and BPA groups before intervention. Results are presented as mean ± standard deviation for continuous variables and n (%) for categorical variables. *P < 0.05. ^#^ See also Table 2. PEA, pulmonary endarterectomy; BPA, balloon pulmonary angioplasty; BMI, body mass index; NT-proBNP, N-terminal pro-B-type natriuretic peptide; 6MWD, 6-min walk distance; WHO-FC, world health organization functional class; RV, diameter of right ventricle (basal); RV/LV, right ventricular-to-left ventricular ratio; EPASP, estimated pulmonary artery systolic pressure; TAPSE, tricuspid annular plane systolic excursion; S’, tricuspid systolic velocity; LVEDD, left ventricular end diastolic diameter; PA, diameter of pulmonary artery; LVEF, left ventricular ejection fraction; FEV1, forced expiratory volume in the 1 s; FVC, forced vital capacity; MVV, maximum voluntary ventilation; DLCO, diffusion capacity of the lungs for carbon monoxide; DLCO%, the percentage of the predicted DLCO; VA, alveolar ventilation; MMEF, maximum mid-expiratory flow; mPAP, mean pulmonary artery pressure; PVR, pulmonary vascular resistance; CO, cardiac output; CI, cardiac index. PCWP, pulmonary capillary wedge pressure.

**TABLE 2:** The oxygen pathway parameters in CTEPH patients before PEA and BPA.

Characteristics	PEA (*n* = 24)	BPA (*n* = 46)	*P*-value
FEV1%, %	84.9 ± 17.9	89.3 ± 20.0	0.244
FVC%, %	100.6 ± 16.0	102.3 ± 19.3	0.715
MMEF%, %	45.1 ± 18.0	47.7 ± 22.0	0.620
MVV%, %	67.0 ± 15.2	66.9 ± 14.1	0.970
VA%, %	94.0 ± 11.5	93.8 ± 14.2	0.968
VA/CO	1.65 ± 0.64	1.56 ± 0.45	0.479
DLO_2_, mmol/min/kPa	7.53 ± 1.70	7.18 ± 1.75	0.426
PalvO_2_, mmHg	103.0 ± 7.1	104.9 ± 6.1	0.248
PaO_2_, mmHg	66.8 ± 14.1	61.3 ± 7.9	0.088
SaO_2_, %	92.5 ± 3.6	91.8 ± 3.0	0.443
SmvO_2_, %	65.3 ± 8.3	65.5 ± 8.2	0.901
CO, L/min	3.46 ± 1.39	3.34 ± 1.09	0.689
CaO_2_, ml/dl	18.8 ± 2.3	17.6 ± 2.5	0.053
DO_2_, ml/min	643.5 ± 251.6	584.1 ± 193.7	0.279
EO_2_, ml/min	177.5 ± 40.4	156.3 ± 45.3	0.061

Each step of the oxygen pathway in CTEPH patients was compared between the PEA and BPA groups before intervention. Results are presented as mean ± standard deviation for continuous variables and n (%) for categorical variables. PEA, pulmonary endarterectomy; BPA, balloon pulmonary angioplasty; FEV1%, the percentage of the forced expiratory volume in the 1 s; FVC%, the percentage of the forced vital capacity; MMEF%, the percentage of the predicted maximum mid-expiratory flow; MVV%, the percentage of the predicted maximum voluntary ventilation; VA%, the percentage of the predicted alveolar ventilation; VA/CO, alveolar ventilation-to-cardiac output ratio; DLO_2_, diffusion capacity of the lungs for oxygen; PalvO_2_, alveolar partial pressure of oxygen; PaO_2_, partial pressure of oxygen in the radial artery; SaO_2_, saturation of oxygen in the radial artery; SmvO_2_, mixed venous oxygen saturation; CO, cardiac output; CaO_2_, arterial oxygen content; DO_2_, oxygen delivery; EO_2_, oxygen extraction.

Impairments were found at each step of the oxygen pathway in patients with CTEPH (Table 2), with no significant differences between the PEA and BPA groups. The MVV% and MMEF% of the CTEPH patients were below normal levels. Lung ventilation-to-lung perfusion was mismatched. A decreased DLO_2_ was speculated to exist in patients with CTEPH, based on less than 80% of DLCO% (Table 1). Blood gas analysis before PEA and BPA showed comparably decreased PaO_2_ (66.8 ± 14.1 vs. 61.3 ± 7.9 mmHg, *P* = 0.088) and SaO_2_ (92.5 ± 3.6 vs. 91.8 ± 3.0%, *P* = 0.443). Similar results were found in oxygen saturation in the pulmonary artery (SmvO_2_) (65.3 ± 8.3 vs. 65.5 ± 8.2%, *P* = 0.901). Comparably decreased CaO_2_ (18.8 ± 2.3 vs. 17.6 ± 2.5 ml/dl, *P* = 0.053) was found in both groups, where the normal value is 20 ml/dl (20). Before the intervention, patients in the PEA group had similar oxygen extraction in peripheral tissue as those in the BPA group (177.5 ± 40.4 vs. 156.3 ± 45.3 ml/min, *P* = 0.061).

### Follow-up

As is shown in Table 3, there were no significant differences in the time since the last intervention among the two groups [330 days (range: 108–1064) in the PEA group and 318 days (range: 41–967) in the BPA group, *P* = 0.836]. Patients in the BPA group attempted an average of 4.0 ± 1.5 sessions of BPA (range: 2–8). Fourteen (30.4%) patients in the BPA group and 2 (8.3%) patients in the PEA group received targeted medications for pulmonary hypertension after the procedure, which was significantly different between the groups (*P* = 0.037).

**TABLE 3:** Comparison between the PEA and BPA groups after intervention.

Characteristics	PEA (*n* = 24)	BPA (*n* = 46)	*P*-value
Time since the last intervention, d	330 (108–1064)	318 (41–967)	0.836
BPA sessions	–	4.0 ± 1.5	–
PH-targeted medication	2 (8.3%)	14 (30.4%)	0.037*
ERA	1 (4.2%)	3 (6.5%)	1.0
PDE5 inhibitors	0 (0.0%)	3 (6.5%)	0.546
Riociguat	1 (4.2%)	8 (17.4%)	0.151
**Functional parameters**			
Change in 6MWD, m	97 ± 106	64 ± 84	0.171
Change in NT-proBNP, pg/ml	−1095 ± 2033	−816 ± 1288	0.544
Change in WHO-FC	−1.6 ± 0.9	−0.9 ± 0.7	0.001*
Change in hemoglobin, g/dl	−1.94 ± 2.63	−0.27 ± 1.61	0.008*
**Echocardiogram**			
Change in RV, mm	−7.5 ± 7.0	−5.4 ± 6.0	0.208
Change in RV/LV	−0.39 ± 0.29	−0.33 ± 0.36	0.540
Change in EPASP, mmHg	−36.1 ± 26.3	−18.9 ± 22.2	0.047*
Change in TAPSE, mm	−3.2 ± 3.9	1.8 ± 3.7	<0.001*
Change in S’, cm/s	−1.6 ± 3.1	0.8 ± 2.6	0.002*
Change in LVEDD, mm	2.1 ± 4.6	3.0 ± 4.5	0.451
Change in PA, mm	−2.6 ± 4.3	0.9 ± 4.2	0.123
Change in LVEF, %	−2.0 ± 5.6	−2.1 ± 6.9	0.956
Pulmonary function test^#^			
Change in FEV1, L	−0.08 ± 0.36	0.08 ± 0.20	0.053
Change in FVC, L	−0.17 ± 0.45	0.12 ± 0.28	0.002*
Change in MVV, L/min	−2.1 ± 11.2	1.4 ± 7.5	0.142
Change in DLCO, mmol/min/kPa	−0.44 ± 0.66	0.04 ± 0.67	0.006*
Change in DLCO%, %	−5.8 ± 7.1	0.6 ± 8.7	0.003*
Change in VA, L	−0.16 ± 0.52	0.03 ± 0.37	0.126
Change in MMEF, L/s	0.02 ± 0.47	0.01 ± 0.37	0.920
**Hemodynamics**			
Change in mPAP, mmHg	−18.2 ± 11.3	−13.9 ± 8.9	0.079
Change in PVR, woods	−8.1 ± 5.0	−5.2 ± 4.9	0.027*
Change in CO, L/min	1.00 ± 1.61	0.36 ± 1.05	0.051
Change in CI, L/min/m^2^	0.57 ± 0.86	0.20 ± 0.57	0.055

After intervention, follow-up duration, medication treatments, and changes in functional, echocardiographic, pulmonary functional, and hemodynamic parameters in CTEPH patients were compared between the PEA and BPA groups. Results are presented as mean ± standard deviation for continuous variables and n (%) for categorical variables, *P < 0.05. ^#^ See also Table 4. CTEPH, chronic thromboembolic pulmonary disease; PEA, pulmonary endarterectomy; BPA, balloon pulmonary angioplasty; ERA, Endothelin receptor antagonists; PDE5, Phosphodiesterase-5; NT-proBNP, N-terminal pro-B-type natriuretic peptide; 6MWD, 6-min walk distance; WHO-FC, world health organization functional class; RV, diameter of right ventricle (basal); RV/LV, right ventricular-to-left ventricular ratio; EPASP, estimated pulmonary artery systolic pressure; TAPSE, tricuspid annular plane systolic excursion; S’, tricuspid systolic velocity; LVEDD, left ventricular end diastolic diameter; PA, diameter of pulmonary artery; LVEF, left ventricular ejection fraction; FEV1, forced expiratory volume in the first second; FVC, forced vital capacity; MVV, maximum voluntary ventilation; DLCO, diffusion capacity of the lungs for carbon monoxide; DLCO%, the percentage of the predicted DLCO; VA, alveolar ventilation; MMEF, maximum mid-expiratory flow; mPAP, mean pulmonary artery pressure; PVR, pulmonary vascular resistance; CO, cardiac output; CI, cardiac index.

**TABLE 4:** Change in the oxygen pathway parameters after PEA and BPA.

Characteristics	PEA (*n* = 24)	BPA (*n* = 46)	*P*-value
Change in FEV1%, %	−3.4 ± 12.7	3.8 ± 8.7	0.006*
Change in FVC%, %	−5.5 ± 13.0	4.2 ± 9.9	0.001*
Change in MMEF%, %	0.2 ± 12.8	0.7 ± 12.3	0.862
Change in MVV%, %	−2.3 ± 9.7	2.5 ± 6.5	0.023*
Change in VA%, %	−3.2 ± 10.5	−0.1 ± 7.8	0.160
Change in VA/CO	−0.48 ± 0.53	−0.17 ± 0.41	0.016*
Change in DLO_2_, mmol/min/kPa	−0.54 ± 0.82	0.05 ± 0.82	0.006*
Change in PalvO_2_, mmHg	−1.9 ± 7.3	−4.9 ± 8.2	0.143
Change in PaO_2_, mmHg	6.9 ± 15.9	3.3 ± 10.4	0.263
Change in SaO_2_, %	2.1 ± 4.3	0.6 ± 3.7	0.126
Change in SmvO_2_, %	2.8 ± 10.5	3.9 ± 8.0	0.651
Change in CO, L/min	1.00 ± 1.61	0.36 ± 1.05	0.051
Change in CaO_2_, ml/dl	−2.0 ± 3.5	−0.3 ± 2.2	0.039*
Change in DO_2_, ml/min	112.7 ± 321.6	46.4 ± 191.6	0.293
Change in EO_2_, ml/min	25.8 ± 68.1	−7.8 ± 42.7	0.016*

Change in each step of the oxygen pathway in CTEPH patients was compared between the PEA and BPA groups after intervention. Results are presented as mean ± standard deviation for continuous variables and n (%) for categorical variables, *P < 0.05. PEA, pulmonary endarterectomy; BPA, balloon pulmonary angioplasty; FEV1%, the percentage of the forced expiratory volume in the 1 s; FVC%, the percentage of the forced vital capacity; MMEF%, the percentage of the predicted maximum mid-expiratory flow; MVV%, the percentage of the predicted maximum voluntary ventilation; VA%, the percentage of the predicted alveolar ventilation; VA/CO, alveolar ventilation-to-cardiac output ratio; DLO_2_, diffusion capacity of the lungs for oxygen; PaO_2_, partial pressure of oxygen in the radial artery; PalvO_2_, alveolar partial pressure of oxygen; SaO_2_, saturation of oxygen in the radial artery; SmvO_2_, mixed venous oxygen saturation; CO, cardiac output; CaO_2_, arterial oxygen content; DO_2_, oxygen delivery; EO_2_, oxygen extraction.

After the intervention, patients with CTEPH had a significant improvement in cardiac function, illustrated by the improvements in 6-min working distance (6MWD), N-terminal pro-B-type natriuretic peptide (NT-proBNP), world health organization functional class (WHO-FC), and smaller right ventricle (Supplementary Figures 1A–C, 2A–H and Supplementary Table 1). Patients in the PEA group had significantly more improvement in their WHO-FC than those in the BPA group (−1.6 ± 0.9 vs. −0.9 ± 0.7, *P* = 0.001; Table 3). Significantly more reduction in the estimated pulmonary artery systolic pressure (EPASP) (−36.1 ± 26.3 vs. −18.9 ± 22.2 mmHg, *P* = 0.047) were found in PEA group than BPA group. Interestingly, compared with an increase of tricuspid annular plane systolic excursion (TAPSE) (from 17.4 ± 2.8 to 18.9 ± 2.8 mm, *P* = 0.003) and tricuspid systolic velocity (S’) (from 10.6 ± 2.1 to 11.4 ± 2.5 cm/s, *P* = 0.046) in the BPA group, those in the PEA group decreased (from 16.3 ± 3.3 to 12.8 ± 2.8 mm, *P* = 0.002 for TAPSE; from 10.0 ± 2.7 to 8.0 ± 1.6 cm/s for S’, *P* = 0.034, respectively), which may be associated with the change in the overall motion of the heart post-PEA and other surgery related injury (21, 22).

### Change in the oxygen pathway

**FIGURE 2:**
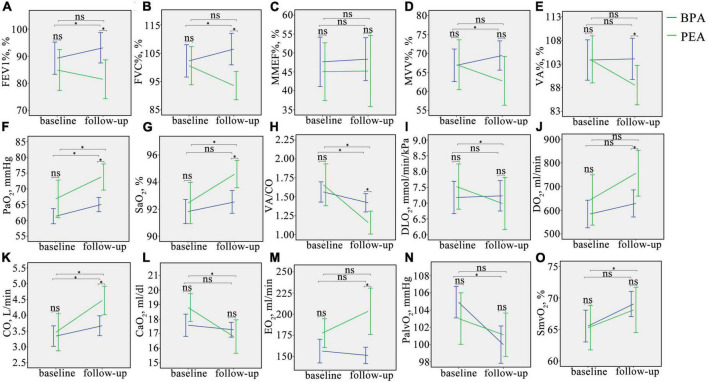
Changes in oxygen pathway parameters in CTEPH patients before and after intervention. **(A–O)**, Change in FEV1%, FVC%, MMEF%, MVV%, VA%, PaO_2,_ SaO_2,_ VA/CO, DLO_2,_ DO_2,_ CO, CaO_2_, EO_2,_ PalvO_2_, and SmvO_2_ in CTEPH patients before and after the intervention. ns, no significant difference, *P < 0.05. FEV1%, the percentage of predicted forced expiratory volume in the 1 s; FVC%, the percentage of predicted forced vital capacity; MMEF%, the percentage of predicted maximum mid-expiratory flow; MVV%, the percentage of predicted maximum voluntary ventilation; VA%, the percentage of predicted alveolar ventilation; PaO_2,_ partial pressure of oxygen in the radial artery; SaO_2,_ saturation of oxygen in the radial artery; VA/CO, alveolar ventilation-to-cardiac output ratio; DLO_2_, diffusion capacity of the lungs for oxygen; DO_2,_ oxygen delivery; CO, cardiac output; CaO_2_, arterial oxygen content; PalvO_2_, alveolar partial pressure of oxygen; EO_2,_ oxygen extraction; SmvO_2,_ mixed venous oxygen saturation.

**FIGURE 3:**
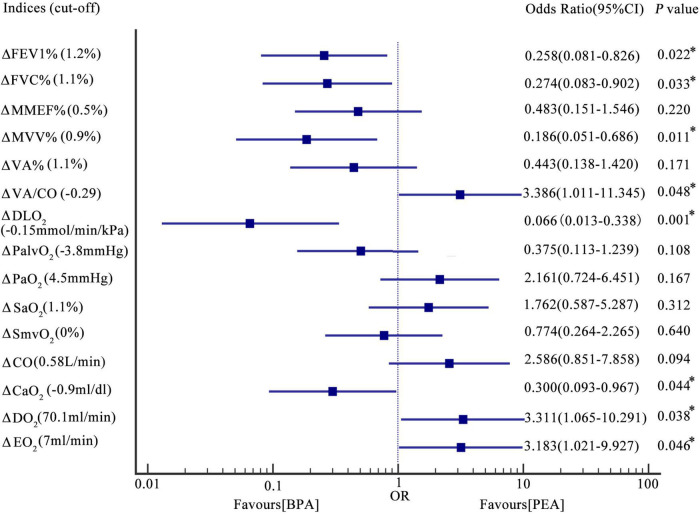
Forest plot-the association between the change in oxygen pathway indices and the interventional method. Results were based on logic regression analysis (see also the statistical analysis section). The incidences of change in FEV1% > 1.2%, FVC% > 1.1%, MVV% > 1.1%, VA/CO < –0.29, DLO_2_ > –0.15 mmol/min/kPa, CaO_2_ > –0.9 ml/dl, DO_2_ > 70.1 ml/min, and EO_2_ > 7 ml/min in patients post-PEA were 0.258, 0.274, 0.186, 3.386, 0.066, 0.300, 3.311, 3.183 times as those in patients post-BPA. Incidences of change in MMEF% > 0.5%, VA% > 1.1%, PalvO_2_ < –3.8 mmHg, PaO_2_ > 4.5 mmHg, SaO_2_ > 1.1%, SmvO_2_ > 0%, and CO > 0.58 L/min were comparable between patients post-PEA and BPA. *P < 0.05. Δ, change in the indices. PEA, pulmonary endarterectomy; BPA, balloon pulmonary angioplasty; FEV1%, the percentage of the forced expiratory volume in the 1 s; FVC%, the percentage of the forced vital capacity; MMEF%, the percentage of the predicted maximum mid-expiratory flow; MVV%, the percentage of the predicted maximum voluntary ventilation; VA%, the percentage of the predicted alveolar ventilation; VA/CO, alveolar ventilation-to-cardiac output ratio; DLO_2_, diffusion capacity of the lungs for oxygen; PaO_2_, partial pressure of oxygen in the radial artery; PalvO_2_, alveolar partial pressure of oxygen; SaO_2_, saturation of oxygen in the radial artery; SmvO_2_, mixed venous oxygen saturation; CO, cardiac output; CaO_2_, arterial oxygen content; DO_2_, oxygen delivery; EO_2_, oxygen extraction.

Patients with CTEPH treated with BPA have improved lung ventilation, including FEV1%, FVC%, and MVV%, while those treated with PEA have a decreasing trend (Figures 2A–E and Supplementary Table 1). The change in FEV1% (−3.4 ± 12.7 vs. 3.8 ± 8.7%, *P* = 0.006), FVC% (−5.5 ± 13.0 vs. 4.2 ± 9.9%, *P* = 0.001), and MVV% (−2.3 ± 9.7% vs. 2.5 ± 6.5%, *P* = 0.023) were significantly different between the PEA and BPA groups (Table 4 and Figure 3). Both MMEF% and VA% in patients treated with PEA or BPA showed no significant change.

In this study, we used PaO_2_ and SaO_2_ to assess the efficacy of pulmonary gas exchange. Both lung diffusion capacity and ventilation/perfusion can have an impact on lung gas exchange. Patients post-PEA had a significant higher PaO_2_ than those post-BPA (73.8 ± 9.8 vs. 65.0 ± 7.5 mmHg, *P* = 0.022) (Figure 2F and Supplementary Table 1). Meanwhile, patients in the PEA group had a significant increase in SaO_2_ (from 92.5 ± 3.6 to 94.6 ± 2.4%, *P* = 0.022) (Figure 2G and Supplementary Table 1), while those in the BPA group had no change in SaO_2_, which could be explained by a better improvement of VA/CO after PEA (−0.48 ± 0.53 vs. −0.17 ± 0.41, *P* = 0.016) (Table 4 and Figures 2H, 3). Interestingly, patients in the PEA group had a significant decrease in DLO_2_ (from 7.53 ± 1 to 6.99 ± 1.95 mmol/min/kPa, *P* = 0.004), while those in the BPA group had no change (Figure 2I; Supplementary Table 1 and Table 4).

The oxygen delivery (DO_2_) in patients after PEA was significantly higher than that in patients treated with BPA (756.3 ± 229.1 vs. 628.8 ± 188.5 ml/min, *P* = 0.016) (Figure 2J and Supplementary Table 1), while a comparable improvement in cardiac output was found among the PEA and BPA groups (1.00 ± 1.61 vs. 0.36 ± 1.05 L/min, *P* = 0.051) (Table 4 and Figures 2K, 3). Compared with patients post-BPA, those post-PEA had a significant decrease in the arterial oxygen content (−2.0 ± 3.5 vs. −0.3 ± 2.2 ml/dl, *P* = 0.039) (Table 4 and Figures 2L, 3).

Although the oxygen extraction of peripheral tissues (EO_2_) at rest in CTEPH patients had no statistically significant change after PEA and BPA (from 177.5 ± 40.4 to 203.3 ± 64.8 ml/min for PEA, *P* = 0.076 and from 156.3 ± 45.3 to 151.2 ± 31.9 ml/min for BPA, *P* = 0.243, respectively) (Figure 2M and Supplementary Table 1), patients treated with PEA had a significant increase in change of oxygen extraction than those treated with BPA (25.8 ± 68.1 vs. −7.8 ± 42.7 ml/min, *P* = 0.016) (Table 4 and Figure 3). In addition, there were no significant difference in the PalvO_2_ (*P* = 0.507, Figure 2N) and the SmvO_2_ (*P* = 0.611, Figure 2O) after the interventions between the PEA and BPA groups.

## Discussion

**FIGURE 4:**
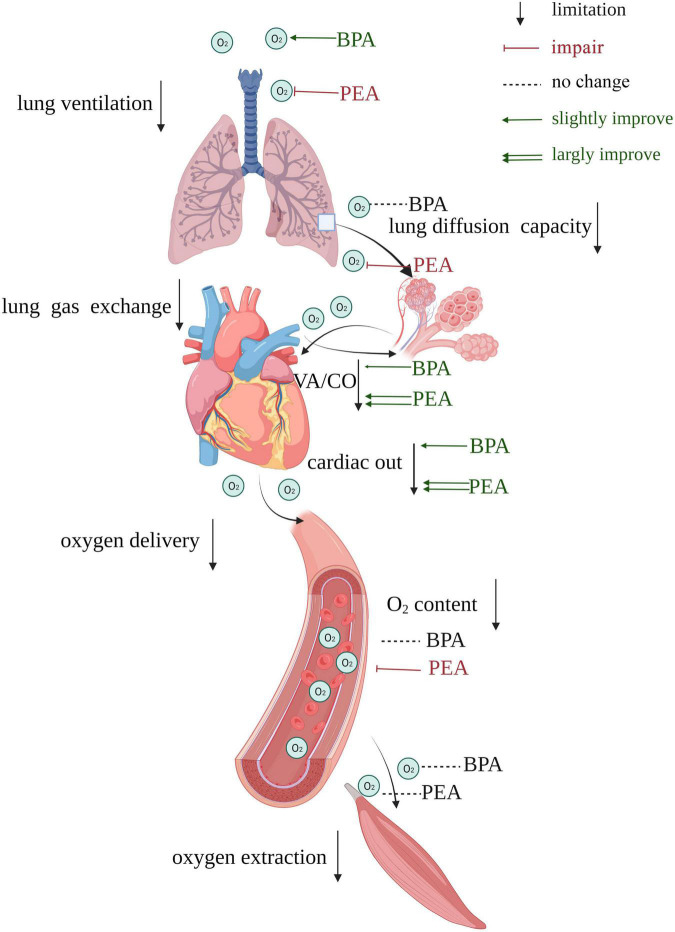
Summary of the effects of PEA versus BPA on the oxygen pathway of patients with CTEPH. Impairments exit in both lung ventilation, lung gas exchange, oxygen delivery, and oxygen extraction. BPA could improve lung ventilation, while PEA causes damage to it. VA/CO gets more improvement in patients receiving PEA than those receiving BPA. PEA impairs lung diffusion capacity, while BPA does not affect it. The cardiac output of patients receiving PEA gets more improvement than those receiving BPA, and PEA could reduce the oxygen content. There is no significant change in oxygen extraction after PEA or BPA. VA/CO, alveolar ventilation-to-cardiac output ratio.

We retrospectively collected data on CTEPH patients receiving PEA or BPA in our center to assess and compare each step of the oxygen pathway before and after intervention and between these two interventions. Such comparisons had not been performed in previous studies. We found that patients with CTEPH had multiple limitations in the oxygen pathway (Table 2), and partial amelioration could be achieved after PEA or BPA. Interestingly, the oxygen pathway of CTEPH patients responded differently to PEA and BPA. Patients post-PEA showed better lung gas exchange and oxygen delivery, while patients post-BPA had better lung ventilation. Although no significant change in the extraction of oxygen was found in patients after the intervention, patients receiving PEA showed more improvement than those receiving BPA. The effects of PEA and BPA on the oxygen pathway of CTEPH patients are shown in Figure 4.

The oxygen uptake involves lung ventilation and lung gas exchange. In this study, we have found that the dysfunction of the small airway in lung ventilation in a single breath (decreased maximum mid-expiratory flow) did not cause insufficient lung ventilation (alveolar ventilation was about 94% of predicted) in patients with CTEPH (Table 2). Maximum mid-expiratory flow (MMEF) is defined as the maximum expiratory flow between 25 and 75% of the FVC and is used to assess small airway obstruction (23). Small airway function is rarely investigated in patients with CTEPH and the MMEF of patients in this study was less than 50% of the predicted value. One previous study has found a potential diagnostic and prognostic value of MMEF in patients with respiratory symptoms without chronic obstructive pulmonary disease (24). In our study, the MMEF was strongly correlated with MVV (*r* = 0.831, *P* < 0.001) in patients with CTEPH. The CTEPH patients of this study showed a slight decrease in maximum voluntary ventilation (∼66% of predicted, Table 2), which reflects a decrease in the respiratory ventilation reserve in CTEPH. It is possible that a decrease in MMEF might contribute to the decrease of MVV. For spirometry, FVC and FEV1 are the two most important parameters (25). The FVC in CTEPH patients was normal in this study (Table 1), consistent with previous studies (26, 27). Compared with a decreased FEV1 in previous studies on CTEPH patients, in our study, the FEV1 was normal. The possible decrease in FEV1 in CTEPH may be associated with airway diseases (25). DLCO is used to assess the diffusion capacity of the lung. In our study, the DLCO in patients with CTEPH was nearly 70% of the predicted value, consistent with the results of previous studies (13, 14). Previous pathological studies have reported frequent presentation of the thickened alveolar wall and deficient angiogenesis in the lungs of CTEPH patients (28), suggesting that the abnormalities of both the alveolar membrane and pulmonary capillary might contribute to the decrease of DLCO in CTEPH. Howden et al. have reported a decrease in oxygen delivery in CTEPH patients at peak exercise (15), and the mean oxygen delivery at rest was < 645 ml/min in CTEPH patients in our study, which we think was below the normal level, and the reasons for which were as follows: (i) The oxygen delivery is determined by cardiac output and arterial oxygen content, which could be affected by any pathophysiology with an impact on these indices (19); (ii) The arterial oxygen content is a parameter derived from SaO_2_ and PaO_2_ (19); and (iii) The cardiac output index (< 1.9 L/min/m^2^), SaO_2_ (< 93%), and PaO_2_ (< 67 mmHg) in CTEPH patients were all below normal levels in this study. The oxygen extraction of patients with CTEPH in our study was less than 180 ml/min (reference value for adults: 250 ml/min) (29), indicating a secondary impairment of oxygen use in the periphery in patients with CTEPH. The mitochondrial oxidative phosphorylation capacity is equal to 1.8 times the uptake of oxygen (15). The mitochondrial oxidative phosphorylation capacity was not calculated in this study, because the cardiopulmonary excise test was not routinely tested in our center. Nevertheless, Gimenez et al. demonstrated that the uptake of oxygen was strong corrected with MVV (*r* = 0.765, *P* < 0.001) (30), and the MVV of CTEPH patients was below the normal level in our study, so it was reasonable to speculate that the mitochondrial oxidative phosphorylation capacity might be decreased.

Chronic thromboembolic pulmonary hypertension patients treated with PEA had a worsened lung diffusion capacity, while patients with CTEPH treated with BPA had an improved lung ventilation. Previous studies have revealed that the lung diffusion capacity in CTEPH patients decreased in the short term (3 weeks) after PEA (14), and could persist for more than 1 year (31), which were consistent with our study (Table 2). Compared with the deterioration of the lung diffusion capacity in CTEPH patients treated with PEA, the impaired lung diffusion capacity was not changed (*P* = 0.679, Supplementary Table 1) in CTEPH patients treated with BPA in both our study and previous studies (26, 32). The reason for the different effects of the two interventions on lung diffusion capacity was likely due to the high reperfusion pulmonary edema (RPE) caused by a rapid decrease of pulmonary hypertension after PEA. The occurrence of RPE was more than 50% in recent PEA studies (33, 34), compared with less than 10% in BPA (32, 35). Another reason for the decrease of lung diffusion capacity after PEA might be mechanical ventilation complications, leading to alveolar damage, possibly attributable to an increase in surfactant protein type B (34). Of note, we used uncorrected DLCO in this study, which could be another reason for the decline in DLCO for patients post-PEA (36). Interestingly, Akizuki et al. found that the lung diffusion capacity in different lung fields responded differently to BPA (26). In their study, the diffusion capacity in the upper-middle lung field in CTEPH patients was improved after BPA, while that in the upper lung field was decreased. Although the lung diffusion capacity of patients with CTEPH was not improved by PEA or BPA in our study, a mild improvement at peak exercise 6 months after intervention [PEA (*n* = 8) and BPA (*n* = 2)] was found in a small prospective study (15). After the intervention, the change in FVC in patients receiving BPA was significantly different from that in patients receiving PEA. The negative trend in FVC in patients post-PEA could be due to the major cardiothoracic surgery they underwent. An increase of FVC in patients receiving BPA was significantly negatively correlated with the change in PVR (*r* = −0.44, *P* < 0.01) in a report from Takei et al. (37). This indicated that some unknown factors, in addition to reperfusion of the pulmonary artery, might be involved in the improvement of pulmonary function in CTEPH patients after BPA. Moreover, the change in MVV% was significantly different between patients receiving BPA and PEA (−2.3 ± 9.7% vs. 2.5 ± 6.5%, *P* = 0.023), and was significantly correlated with the change in FVC (*r* = 0.803, *P* < 0.001).

Despite better lung ventilation in patients receiving BPA than those receiving PEA, there was no significant difference in the change in VA (*P* = 0.126). It seemed that PEA patients had a reduction in anatomical dead space due to the thoracic surgery, which requires further research. On the other hand, patients treated with PEA in this study tended to show more improvement in lung gas exchange, which could be explained by a better VA/CO (1.16 vs. 1.42, *P* = 0.010), attributable to a higher cardiac output (4.47 ± 1.06 L/min vs. 3.67 ± 1.04 L/min, *P* = 0.004) after PEA. Moreover, Pearson’s correlation analysis revealed that the change in VA/CO was correlated with the change in PaO_2_ (*r* = −0.377, *P* = 0.003) and SaO_2_ (*r* = −0.410, *P* = 0.001). Those evidences indicated that PEA may be superior to BPA in term of vascular recanalization, which contributed to more reduction in physiological dead space.

In our study, the oxygen delivery in patients treated with PEA was significantly higher than that in patients treated with BPA (*P* = 0.016), attributable to a better cardiac output after the intervention. Based on the evidence that the cardiac output was significantly improved, even superior to BPA, in inoperable CTPEH patients who received riociguat (38), combination therapy of BPA and riociguat could be prescribed to better improve the oxygen delivery in inoperable CTEPH patients. Interestingly, despite an improvement of SaO_2_, the oxygen content decreased in patients post-PEA (*P* = 0.011), which can be explained by the decrease of hemoglobin after PEA (from 14.8 ± 1.9 to 12.9 ± 2.0 g/dl, *P* = 0.001, Supplementary Table 1). This phenomenon was also found in the paper from Howden et al. (15) possibly attributable to a decrease of erythropoietin resulting from an improvement of hypoxia (39, 40), or loss of hemoglobin caused by the intervention (41).

According to Howden et al., the oxygen extraction by peripheral tissues at peak exercise was normal in patients with CTEPH (15). In spite of this, skeletal muscle dysfunction existed reportedly in CTEPH patients (42), which indicates the decrease in other nutrients supply due to the reduced cardiac output, may result in the muscle dysfunction. In our study, the oxygen extraction at rest showed no change after intervention. However, there was a significant difference in the change in oxygen extraction among patients treated with PEA versus BPA (Table 4 and Figure 3), where patients treated with PEA had a better performance in oxygen extraction than those receiving BPA. The oxygen extraction is comprised of oxygen transporting from capillary to cells and oxygen utilization by the mitochondria (19). Therefore, the higher oxygen extraction in PEA patients could be partly due to change in oxygen transporting from capillary to cells, resulting from significant improvement in oxygen delivery. Interestingly, a normal extraction of peripheral tissues was found at rest and peak exercise, and the muscle diffusion capacity of oxygen was below the normal level in their study, which suggested a potential benefit of cardiopulmonary rehabilitation in CTEPH patients.

Cardiopulmonary rehabilitation could improve the cardiac output of patients with CTEPH, which in turn could improve the mismatched lung ventilation/perfusion and oxygen delivery (43). In addition, based on the evidence that patients with heart failure could improve their oxygen extraction after exercise training (44, 45), the oxygen extraction in CTEPH patients is likely to benefit from cardiopulmonary rehabilitation. Although no previous study has been designed to investigate the effect of cardiopulmonary rehabilitation on lung diffusion capacity, the lung diffusion capacity in CTEPH patients don’t seem to benefit from cardiopulmonary rehabilitation based on the evidence that no superior results have been found in other respiratory diseases (sarcoidosis, pulmonary fibrosis and cystic fibrosis) in previous research (46–48). Riociguat and treprostinil have been approved for patients with CTEPH and could increase the cardiac output (49, 50). Other PH-targeted medications, such as phosphodiesterase-5 inhibitors and macitentan, have the same effect (51, 52). Of note, riociguat could increase the area of lung gas exchange through its pro-angiogenic function (53). However, in our study, there were no significant differences in change in lung gas exchange (0.00 ± 0.69 vs. 0.07 ± 0.89 mmol/min/kPa, *P* = 0.786 for change in DLO_2_; 0.6 ± 1.8 vs. 0.6 ± 4.2%, *P* = 0.995 for change in SaO_2_, respectively), oxygen delivery (0.74 ± 0.7 vs. 0.21 ± 1.13 L/min, *P* = 0.129 for change in CO; 91.7 ± 128.4 vs. 28.9 ± 210.3 ml/min, *P* = 0.341 for change in DO_2_, respectively), and oxygen extraction (0.2 ± 50.4 vs. −11.0 ± 39.8 ml/min, *P* = 0.451) between patients treated with and without targeted medications after BPA (Supplementary Table 2). On the contrary, patients treated with targeted medications after BPA had lower improvements in FEV1% (−0.1 ± 7.3 vs. 5.5 ± 8.8%, *P* = 0.041), MMEF% (−5.5 ± 7.2 vs. 3.4 ± 13.1%, *P* = 0.022), and MVV% (−0.5 ± 5.3 vs. 3.8 ± 6.7%, *P* = 0.043) than those without receiving targeted medications. This phenomenon could be explained by the fact that patients with higher pulmonary hypertension were more likely to receive targeted medications according to the contemporary guideline (54). And patients post-BPA, who were treated with targeted medications after the last session of intervention, had higher mean pulmonary artery pressure (29.9 ± 7.6 vs. 25.1 ± 5.9 mmHg, *P* = 0.030) than those without receiving targeted medications in our study. Many CTEPH patients may have mixed vascular lesions with both proximal and distal lesions. In term of reducing lung ventilation impairment post-PEA, combination therapy of PEA and BPA could be an option for those patients according to our study. Another option could be minimally invasive PEA surgery, which is performed through mini-anterior thoracotomy instead of sternotomy (55). Some CTEPH patients still showed persistent pulmonary hypertension even after several BPA sessions (56), therefore it is essential to identify the predictors of hemodynamic response. Zhihong Liu and her colleagues found a baseline DLCO% < 70% and change in DLCO% > 6% could be an unfavorable predictor for BPA (32). According to the research from Tsuji A. et al., preoperative FEV1 was a predictor of residual pulmonary hypertension after BPA in CTEPH patients (56). Moreover, postoperative PaO_2_ was a prognostic predictor of patients post-PEA (57). And mixed venous oxygen saturation was reportedly associated with prognosis of patients post-PEA and improved renal function of patients after BPA (58, 59). Therefore, the oxygen pathway parameters could be used to guide decision-making in the management of CTEPH patients.

This study has several limitations, mainly owing to the retrospective design. The main limitation is that the pulmonary function was tested at the upright position, while the hemodynamics was evaluated at the supine position, which could influence the result of VA/CO (60). Secondly, after all exclusions only 24 patients post-PEA and 46 patients post-BPA remained, which is suggesting potential health worker survivor bias. Thirdly, patients not suitable for PEA may be technically more difficult, and may have a poor risk-benefit because of comorbidities, in spite of a comparable pre-interventional WHO-FC and post-interventional mPAP (24.3 ± 8.5 vs. 26.5 ± 6.7 mmHg, *P* = 0.238, Supplementary Table 2) among the PEA and BPA groups.

In conclusion, in this single-center retrospective study, we evaluated and compared each step of the oxygen pathway in CTEPH patients treated with PEA and BPA. Partial improvements in oxygen limitation could be achieved after PEA and BPA, with key differences in the responses to these treatments. Additional research is warranted to investigate the effect of targeted medications for pulmonary hypertension and the role of the oxygen pathway in the management of CTEPH. Given the cardiopulmonary and peripheral defects in the oxygen pathway, cardiopulmonary rehabilitation can be prescribed to patients treated after PEA and BPA.

## Data availability statement

The original contributions presented in this study are included in the article/Supplementary material, further inquiries can be directed to the corresponding author.

## Ethics statement

The studies involving human participants were reviewed and approved by the Institutional Board and the Ethics Committee of the China-Japan Friendship Hospital (2021-136-K94). The patients/participants provided their written informed consent to participate in this study.

## Author contributions

ZZ had full access to all the data and takes responsibility for its integrity and the data analysis. ZF contributed to the study conception and design, data collection and analysis, and drafting of the manuscript. XT, WX, QG, and JW were involved in the data collection. PY was involved in revising the manuscript. All authors have read and approved the final manuscript.
