# Supplementary material for: Different response of the oxygen pathway in patients with chronic thromboembolic pulmonary hypertension treated with pulmonary endarterectomy versus balloon pulmonary angioplasty
Source: Front Cardiovasc Med. 2022 Sep 27;9:990207. doi: 10.3389/fcvm.2022.990207 (PMC9551285; doi:10.3389/fcvm.2022.990207)
Supplement: Supplementary file 1 [file Table_1.docx]

**Table S1 Characteristics of CTEPH patients before and after PEA and BPA**

| Characteristics | PEA (n=24)  pre-PEA post-PEA | | *P* value^†^ | BPA (n=46)  pre-BPA post-BPA | | *P* value^‡^ | *P* value^§^ | *P* value^\|\|^ |
| --- | --- | --- | --- | --- | --- | --- | --- | --- |
| WHO-FC | | | | | | | | |
| I-II | 9 (37.5%) | 23 (95.8%) | <0.001^*^ | 26 (56.6%) | 42 (91.3%) | <0.001^*^ | 0.208 | 0.654 |
| III-IV | 15 (62.5%) | 1 (4.2%) |  | 20 (43.4%) | 4 (8.7%) |  |  |  |
| 6MWD, m | 367±91 | 455±87 | 0.001^*^ | 383±90 | 446±84 | <0.001^*^ | 0.758 | 0.687 |
| NT-proBNP, pg/ml | 1421±2022 | 326±524 | 0.015^*^ | 1007±1331 | 191±281 | <0.001^*^ | 0.307 | 0.165 |
| Hemoglobin, g/dl | 14.8±1.9 | 12.9±2.0 | 0.001^*^ | 13.9±2.1 | 13.6±1.3 | 0.254 | 0.071 | 0.074 |
| Echocardiogram | | | | | | | | |
| RV, mm | 46.1±6.5 | 38.6±5.6 | <0.001^*^ | 44.7±7.1 | 39.1±5.4 | <0.001^*^ | 0.428 | 0.714 |
| RV/LV | 1.32±0.31 | 0.94±0.17 | <0.001^*^ | 1.34±0.38 | 1.01±0.13 | <0.001^*^ | 0.805 | 0.061 |
| EPASP, mmHg | 80.1±18.2 | 44.7±16.2 | 0.001^*^ | 78.5±19.4 | 57.3±18.2 | <0.001^*^ | 0.768 | 0.051 |
| TAPSE, mm | 16.3±3.3 | 12.8±2.8 | 0.002^*^ | 17.4±2.8 | 18.9±2.8 | 0.003^*^ | 0.176 | <0.001^*^ |
| S’, cm/s | 10.0±2.7 | 8.0±1.6 | 0.034^*^ | 10.6±2.1 | 11.4±2.5 | 0.046^*^ | 0.260 | <0.001^*^ |
| LVEDD, mm | 43.9±5.6 | 45.5±3.2 | 0.038^*^ | 42.2±4.3 | 46.2±4.8 | <0.001^*^ | 0.868 | 0.523 |
| PA, mm | 32.6±6.8 | 29.9±6.1 | 0.009^*^ | 33.3±6.4 | 32.6±6.5 | 0.174 | 0.697 | 0.100 |
| LVEF, % | 69.9±4.9 | 67.9±4.2 | 0.089 | 69.3±5.3 | 67.1±5.8 | 0.043^*^ | 0.641 | 0.584 |
| Pulmonary function test | | | | | | | | |
| FEV1, L | 2.42±0.81 | 2.34±0.84 | 0.271 | 2.21±0.63 | 2.29±0.60 | 0.012^*^ | 0.244 | 0.790 |
| FEV1%, % | 84.9±17.9 | 81.5±17.0 | 0.198 | 89.3±20.0 | 93.1±18.9 | 0.005^*^ | 0.371 | 0.014^*^ |
| FVC, L | 3.48±0.96 | 3.33±0.97 | 0.093 | 3.09±0.78 | 3.32±0.97 | 0.007^*^ | 0.071 | 0.591 |
| FVC%, % | 100.6±16.0 | 93.6±11.3 | 0.060 | 102.3±19.3 | 106.5±18.7 | 0.006^*^ | 0.715 | 0.001^*^ |
| MVV, L/min | 73.4±24.7 | 70.1±25.9 | 0.392 | 67.3±19.4 | 69.1±18.6 | 0.238 | 0.270 | 0.860 |
| MVV%, % | 67.0±15.2 | 62.8±14.5 | 0.284 | 66.9±14.1 | 69.5±12.6 | 0.017^*^ | 0.970 | 0.059 |
| DLCO, mmol/min/kPa | 6.11±1.38 | 5.68±1.59 | 0.004^*^ | 5.83±1.40 | 5.87±1.32 | 0.679 | 0.426 | 0.581 |
| DLCO%, % | 71.8±14.0 | 66.0±13.8 | 0.001^*^ | 74.1±14.2 | 74.6±11.9 | 0.669 | 0.533 | 0.008^*^ |
| VA, L | 5.20±1.11 | 5.00±1.03 | 0.162 | 4.90±0.93 | 4.90±0.93 | 0.542 | 0.251 | 0.251 |
| VA%, % | 94.0±11.5 | 88.5±9.4 | 0.174 | 93.8±14.2 | 93.8±14.2 | 0.926 | 0.968 | 0.040^*^ |
| MMEF, L/s | 1.59±0.83 | 1.61±1.01 | 0.852 | 1.49±0.80 | 1.50±0.74 | 0.889 | 0.639 | 0.617 |
| MMEF%, % | 45.1±18.0 | 45.2±22.2 | 0.952 | 47.7±22.0 | 48.4±19.1 | 0.699 | 0.620 | 0.538 |
| Hemodynamics | | | | | | | | |
| mPAP, mmHg | 42.5±11.1 | 24.3±8.5 | <0.001^*^ | 40.4±9.3 | 26.5±6.7 | <0.001^*^ | 0.394 | 0.238 |
| PVR, woods | 11.9±5.9 | 3.9±2.7 | <0.001^*^ | 10.2±4.4 | 5.1±2.6 | <0.001^*^ | 0.181 | 0.181 |
| CO, L/min | 3.46±1.39 | 4.47±1.06 | 0.006^*^ | 3.34±1.09 | 3.67±1.05 | 0.025^*^ | 0.689 | 0.004^*^ |
| CI, L/min/m^2^ | 1.86±0.75 | 2.43±0.62 | 0.004^*^ | 1.82±0.53 | 2.00±0.52 | 0.021^*^ | 0.807 | 0.007^*^ |
| Oxygen pathway parameters | | | | | | | | |
| FEV1%, % | 84.9±17.9 | 81.5±17.0 | 0.198 | 89.3±20.0 | 93.1±18.9 | 0.005^*^ | 0.244 | 0.014^*^ |
| FVC%, % | 100.6±16.0 | 93.6±11.3 | 0.060 | 102.3±19.3 | 106.5±18.7 | 0.006^*^ | 0.715 | 0.001^*^ |
| MMEF%, % | 45.1±18.0 | 45.2±22.2 | 0.952 | 47.7±22.0 | 48.4±19.1 | 0.699 | 0.620 | 0.538 |
| MVV%, % | 67.0±15.2 | 62.8±14.5 | 0.284 | 66.9±14.1 | 69.5±12.6 | 0.017^*^ | 0.970 | 0.059 |
| VA%, % | 94.0±11.5 | 88.5±9.4 | 0.174 | 93.8±14.2 | 93.8±14.2 | 0.926 | 0.968 | 0.040^*^ |
| VA/CO | 1.65±0.64 | 1.16±0.34 | 0.001^*^ | 1.56±0.45 | 1.42±0.38 | 0.010^*^ | 0.479 | 0.010^*^ |
| DLO_2,_ mmol/min/kPa | 7.53±1.70 | 6.99±1.95 | 0.004^*^ | 7.18±1.75 | 7.23±1.62 | 0.679 | 0.426 | 0.581 |
| PalvO_2,_ mmHg | 103.0±7.1 | 101.1±6.0 | 0.221 | 104.9±6.1 | 100.0±7.2 | <0.001^*^ | 0.248 | 0.507 |
| PaO_2_, mmHg | 66.8±14.1 | 73.8±9.8 | 0.044^*^ | 61.3±7.9 | 65.0±7.5 | 0.040^*^ | 0.088 | <0.001^*^ |
| SaO_2_, % | 92.5±3.6 | 94.6±2.4 | 0.022^*^ | 91.8±3.0 | 92.5±2.8 | 0.273 | 0.443 | 0.003^*^ |
| SmvO_2_, % | 65.3±8.3 | 68.1±8.5 | 0.202 | 65.5±8.2 | 69.1±6.6 | 0.003^*^ | 0.901 | 0.611 |
| CO, L/min | 3.46±1.39 | 4.47±1.06 | 0.006^*^ | 3.34±1.09 | 3.67±1.04 | 0.025^*^ | 0.689 | 0.004^*^ |
| CaO_2_, ml/dl | 18.8±2.3 | 16.8±2.7 | 0.011^*^ | 17.6±2.5 | 17.3±1.7 | 0.370 | 0.053 | 0.450 |
| DO_2_, ml/min | 643.5±251.6 | 756.3±229.1 | 0.099 | 584.1±193.7 | 628.8±188.5 | 0.120 | 0.279 | 0.016^*^ |
| EO_2_, ml/min | 177.5±40.4 | 203.3±64.8 | 0.076 | 156.3±45.3 | 151.2±31.9 | 0.243 | 0.061 | 0.001^*^ |

Functional, echocardiographic, pulmonary functional, hemodynamic, and oxygen pathway parameters in CTEPH patients were compared between the PEA and BPA groups before and after intervention. Results are presented as mean ± standard deviation for continuous variables and n (%) for categorical variables. ^*^*P*<0.05; ^†^pre-PEA vs post-PEA; ^‡^pre-BPA vs post-BPA; ^§^pre-PEA vs pre-BPA; ^||^post-PEA vs post-BPA. List of abbreviations: PEA, pulmonary endarterectomy; BPA, balloon pulmonary angioplasty; NT-proBNP, N-terminal pro-B-type natriuretic peptide; 6MWD, 6-min walk distance; WHO-FC, world health organization functional class; RV, diameter of right ventricle(basal); RV/LV, right ventricular-to-left ventricular ratio; EPASP, estimated pulmonary artery systolic pressure; TAPSE, tricuspid annular plane systolic excursion; S’, tricuspid systolic velocity; LVEDD, left ventricular end diastolic diameter; PA, diameter of pulmonary artery; LVEF, left ventricular ejection fraction; FEV1, forced expiratory volume in the 1s; FEV1%, the percentage of the predicted FEV1; FVC, forced volume capacity; FVC%, the percentage of the predicted FVC; MVV, maximum voluntary ventilation; MVV%, the percentage of the predicted MVV; DLCO, diffusion capacity of the lungs for carbon monoxide; DLCO%, the percentage of the predicted DLCO; VA, alveolar ventilation; VA%, the percentage of the predicted VA; MMEF, maximum mid-expiratory flow; MMEF%, the percentage of the predicted MMEF; mPAP, mean pulmonary artery pressure; PVR, pulmonary vascular resistance; CO, cardiac out; CI, cardiac index; VA/CO, alveolar ventilation-to-cardiac out ratio; DLO_2_, diffusion capacity of the lungs for oxygen; PalvO_2_, alveolar partial pressure of oxygen; PaO_2_, partial pressure of oxygen in radial artery; SaO_2_, saturation of oxygen in radial artery; SmvO_2_, mixed venous oxygen saturation; DO_2_, oxygen delivery; EO_2_,oxygen extraction.
