# Supplementary material for: Different response of the oxygen pathway in patients with chronic thromboembolic pulmonary hypertension treated with pulmonary endarterectomy versus balloon pulmonary angioplasty
Source: Front Cardiovasc Med. 2022 Sep 27;9:990207. doi: 10.3389/fcvm.2022.990207 (PMC9551285; doi:10.3389/fcvm.2022.990207)
Supplement: Supplementary file 2 [file Table_2.docx]

**Table S2 Change in the oxygen pathway parameters after BPA in patients treated with and patients not treated with targeted medications for pulmonary hypertension**

| Characteristics | Targeted Medications+ (n=14) | Targeted Medications- (n=32) | *P* value |
| --- | --- | --- | --- |
| Age, y | 61.0±7.2 | 60.0±9.8 | 0.344 |
| Male | 6(42.9%) | 14(43.8%) | 0.955 |
| BPA sessions | 3.9±1.7 | 4.1±1.4 | *­*0.731 |
| mPAP, mmHg | 29.9±7.6 | 25.1±5.9 | 0.030^*^ |
| Change in FEV1%, % | -0.1±7.3 | 5.5±8.8 | 0.041^*^ |
| Change in FVC%, % | 3.3±11.4 | 4.5±9.3 | 0.701 |
| Change in MMEF%, % | -5.5±7.2 | 3.4±13.1 | 0.022^*^ |
| Change in MVV%, % | -0.5±5.3 | 3.8±6.7 | 0.043^*^ |
| Change in VA%, % | -0.5±10.2 | 0.4±6.7 | 0.744 |
| Change in VA/CO | -0.32±0.35 | -0.11±0.43 | 0.146 |
| Change in DLO_2,_ mmol/min/kPa | 0.00±0.69 | 0.07±0.89 | 0.786 |
| Change in PalvO_2,_ mmHg | -2.0±2.7 | -6.1±9.4 | 0.139 |
| Change in PaO_2_, mmHg | 2.2±5.7 | 3.8±11.9 | 0.565 |
| Change in SaO_2_, % | 0.6±1.8 | 0.6±4.2 | 0.995 |
| Change in SmvO_2_, % | 3.5±9.3 | 4.1±7.6 | 0.804 |
| Change in CO, L/min | 0.74±0.7 | 0.21±1.13 | 0.129 |
| Change in CaO_2_, ml/min | -1.0±1.2 | -0.0±2.4 | 0.198 |
| Change in DO_2_, ml/min | 91.7±128.4 | 28.9±210.3 | 0.341 |
| Change in EO_2_, ml/min | 0.2±50.4 | -11.0±39.8 | 0.451 |

Change in each step of the oxygen pathway was compared between BPA patients with targeted medications (Targeted Medications+) and those without targeted medications (Targeted Medications-) after intervention. Results are presented as mean ± standard deviation for continuous variables and n (%) for categorical variables. ^*^*P*<0.05. List of abbreviations: BPA, balloon pulmonary angioplasty; mPAP, mean pulmonary artery pressure; FEV1%, the percentage of the forced expiratory volume in the 1s; FVC%, the percentage of the forced volume capacity; MMEF%, the percentage of the predicted maximum mid-expiratory flow; MVV%, the percentage of the predicted maximum voluntary ventilation; VA%, the percentage of the predicted alveolar ventilation; VA/CO, alveolar ventilation-to-cardiac out ratio; DLO_2_, diffusion capacity of the lungs for oxygen; PalvO_2,_ alveolar partial pressure of oxygen, PaO_2_, partial pressure of oxygen in the radial artery; SaO_2_, saturation of oxygen in the radial artery; SmvO2, mixed venous oxygen saturation; CO, cardiac out; CaO_2_, arterial oxygen content; DO_2_, oxygen delivery; EO_2_, oxygen extraction.
