# Supplementary figures and images for: Different response of the oxygen pathway in patients with chronic thromboembolic pulmonary hypertension treated with pulmonary endarterectomy versus balloon pulmonary angioplasty
Source: Front Cardiovasc Med. 2022 Sep 27;9:990207. doi: 10.3389/fcvm.2022.990207 (PMC9551285; doi:10.3389/fcvm.2022.990207)

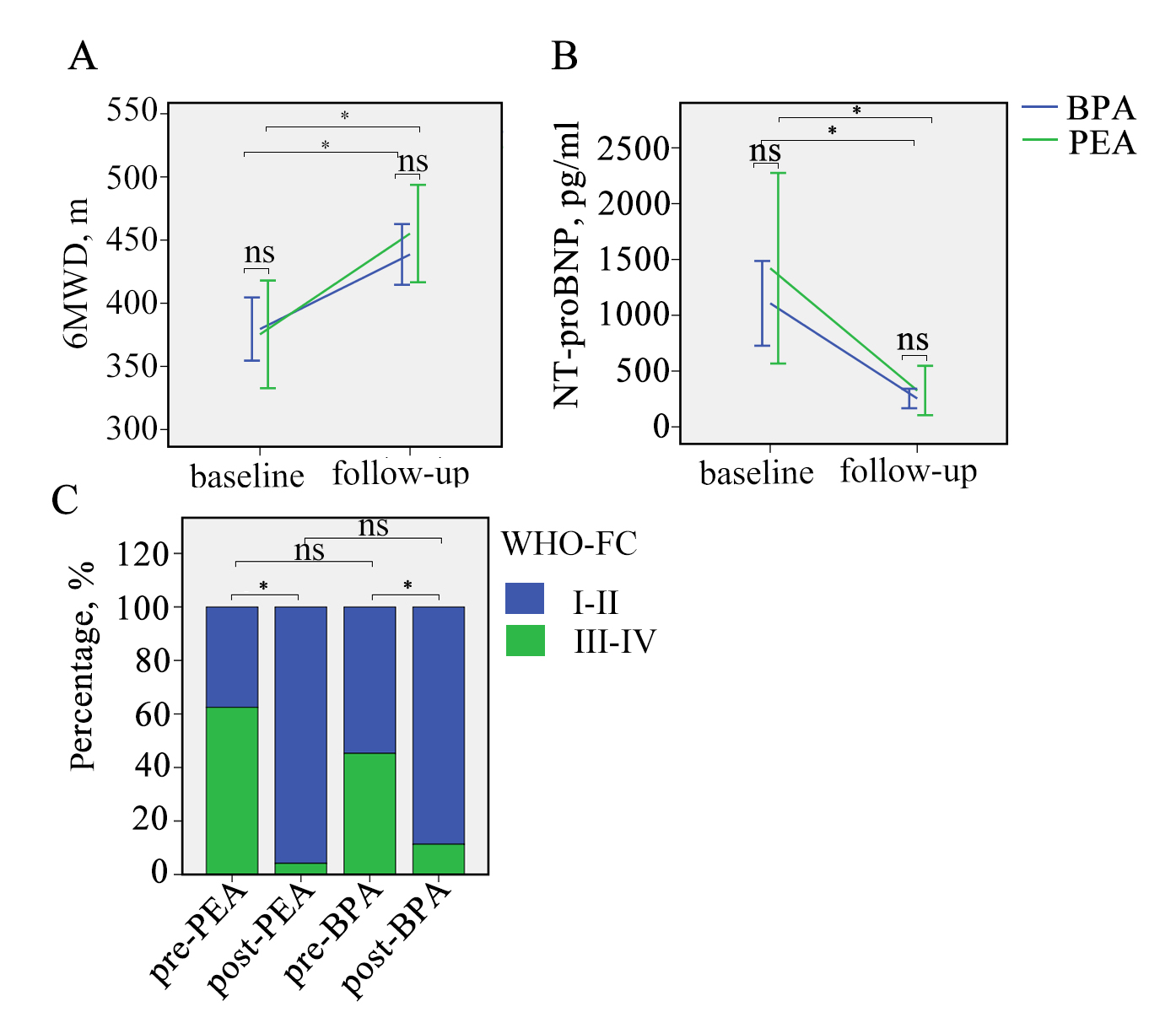

Supplement: Supplementary file 3 [file Image_1.jpeg]

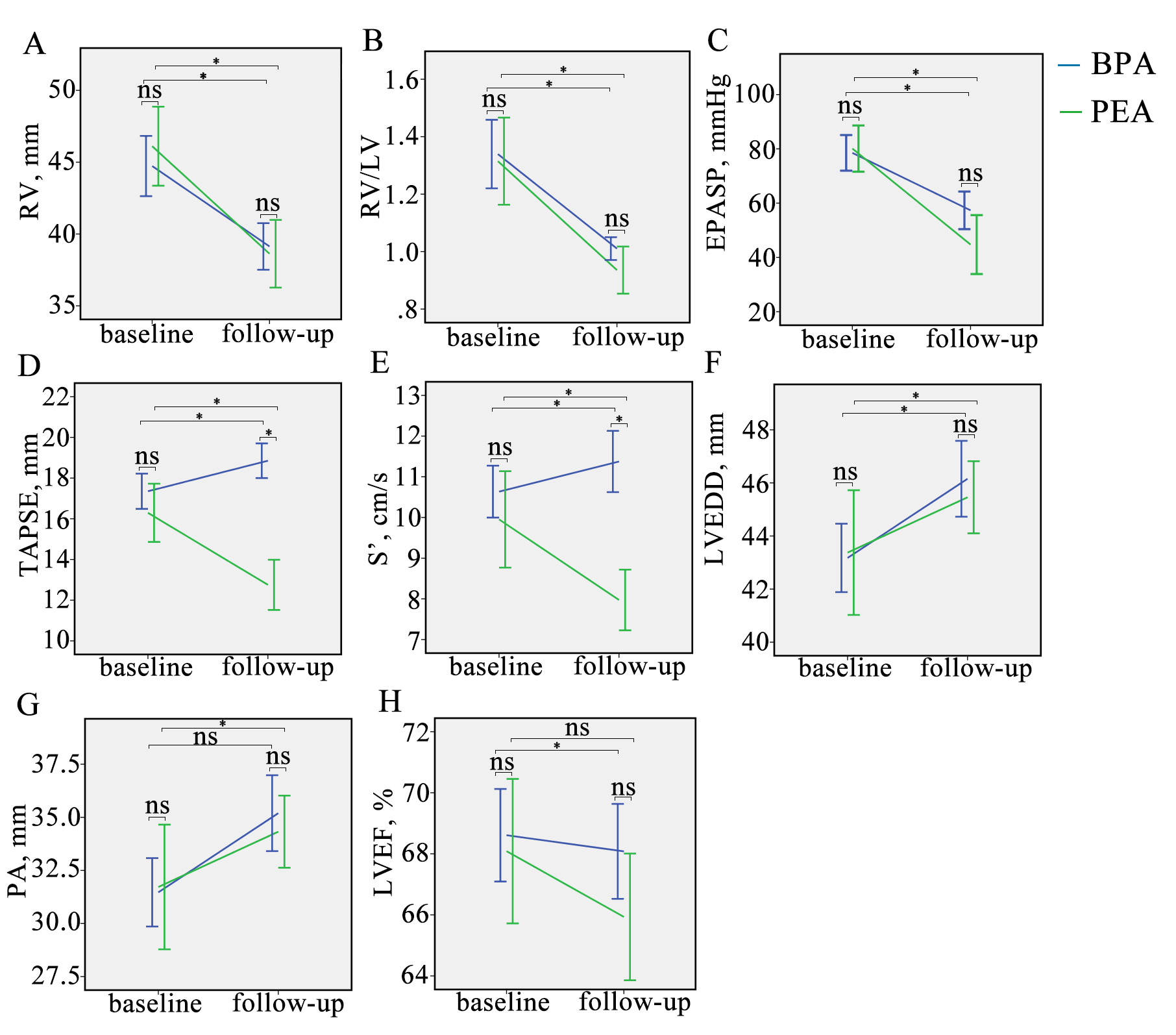

Supplement: Supplementary file 4 [file Image_2.jpeg]
